# Mutation Analysis of 16 Mucolipidosis II and III Alpha/Beta Chinese Children Revealed Genotype-Phenotype Correlations

**DOI:** 10.1371/journal.pone.0163204

**Published:** 2016-09-23

**Authors:** Shuang Liu, Weimin Zhang, Huiping Shi, Fengxia Yao, Min Wei, Zhengqing Qiu

**Affiliations:** 1 Department of Emergency, Peking Union Medical College Hospital, Beijing, China; 2 Clinical Research Laboratory, Peking Union Medical College Hospital, Chinese Academy of Medical Sciences, Beijing, China; 3 Department of Medical Genetics, Institute of Basic Medical Sciences, Beijing, China; 4 Department of Pediatrics, Peking Union Medical College Hospital, Beijing, China; The Ohio State University, UNITED STATES

## Abstract

Mucolipidosis II and III alpha/beta are autosomal recessive diseases caused by mutations in the *GNPTAB* gene which encodes the α and β subunits of the N-acetylglucosamine-1-phosphotransferase. Clinically, mucolipidosis II (MLII) is characterized by severe developmental delay, coarse facial features, skeletal deformities, and other systemic involvement. In contrast, MLIII alpha/beta is a much milder disorder, the symptoms of which include progressive joint stiffness, short stature, and scoliosis. To study the relationship between the genotypes and phenotypes of the MLII and MLIII alpha/beta patients, we analyzed the *GNPTAB* gene in 16 Chinese MLII and MLIII alpha/beta patients. We collected and analyzed the patients’ available clinical data and all showed clinical features typical of MLII or MLIII alpha/beta. Moreover, the activity of several lysosomal enzymes was measured in the plasma and finally the *GNPTAB* gene was sequenced. We detected 30 mutant alleles out of 32 alleles in our patients. These include 10 new mutations (c.99delC, c.118-1G>A, c.523_524delAAinsG, c.1212C>G, c.2213C>A, c.2345C>T, c.2356C>T, c.2455G>T, c.2821dupA, and c.3136-2A>G) and 5 previously reported mutations (c.1071G>A, c.1090C>T, c.2715+1G>A, c.2550_2554delGAAA, and c.3613C>T). The most frequent mutation was the splicing mutation c.2715+1G>A, which accounted for 28% of the mutations. The majority of the mutations reported in the Chinese patients (57%) were located on exon 13 or in its intronic flanking regions.

## Introduction

Mucolipidosis types II (MLII or I-cell disease; MIM# 252500) and III alpha/beta (MLIII alpha/beta, or pseudo-Hurler polydystrophy; MIM# 252600) are autosomal recessive disorders that result from defects in the membrane-bound N-acetylgucosamine-1-phosphotransferase (GLcNAc) [1–2]. This enzyme is responsible for the initial step in the synthesis of the mannose 6-phosphate (M6P) recognition markers on lysosomal enzymes in the Golgi apparatus. Without the M6P recognition markers, the synthesized lysosomal enzymes are secreted from the Golgi to the extracellular space [1]. Therefore, mucolipidosis II/III patients exhibit deficiencies of multiple lysosomal enzymes in many cell types, together with very high enzyme activity levels in extracellular fluids such as plasma and serum [1].

In 1996, Bao et al. confirmed that GlcNAc-1-phosphotransferase was a 540-kDa α2β2γ2 hexameric complex [3]. The α/β subunits are encoded by the *GNPTAB* gene, and the γ subunit is encoded by the *GNPTG* gene [4–6]. The *GNPTAB* gene (MIM# 607840) spans 85 kb of chromosome 12q23.3 and includes 21 exons encoding 1256 amino acids. *GNPTAB* encodes the α/β precursor that is proteolytically cleaved by the site-1 protease in the Golgi apparatus to mature α and β subunits, which is a prerequisite for enzymatic activity [7]. The α/β subunit boundary is located within exon 14. Mutations in *GNPTAB* are known to be responsible for MLII and MLIII alpha/beta. Mutations in the *GNPTG* gene (MIM#252605) cause ML III gamma [4–6].

Clinically, MLII is characterized by the early onset of symptoms that are recognizable at birth and that include severe developmental delay, generalized hypotonia, coarse facial features, gingival hyperplasia, short stature and severe radiological abnormalities. A rapidly progressive clinical course leads to death during the first decade of life [1,8]. In contrast, MLIII is a much milder disorder. Clinically, MLIII alpha/beta and MLIII gamma are both rare diseases and are indistinguishable. The clinical symptoms include progressive joint stiffness, short stature, and scoliosis. Most patients exhibit cardiac valve involvement and experience bone pain and disability due to destruction of the hip joints. The slower clinical course generally results in survival to adulthood [1,9–11].

We analyzed the *GNPTAB* gene in 16 Chinese MLII and MLIII alpha/beta patients who had been diagnosed clinically and biochemically, and we studied the relationships of the obtained genotypes and phenotypes.

## Materials and Methods

### Ethical aspects

We confirm that the study was reviewed and approved by an institutional review board (ethics committee) before the study began. The specific name of the ethics committee that approved our study is Peking Union Medical College Hospital Ethics Review Board. All of the participants provided written informed consent to participate in this study, and the ethics committees approved this consent procedure. We obtained written informed consent from the guardians on behalf of the children enrolled in our study. We have already obtained written consent to publish potentially identifiable information.

### Patients

The 16 unrelated Chinese patients were diagnosed by the Peking Union Medical College Hospital Institutional Review Board between 2006 and 2013. The diagnoses included 8 cases of MLII and 8 cases of MLIII alpha/beta. All of the patients came from nonconsanguineous families. The diagnosis of ML was based on clinical manifestations and lysosomal enzyme activities in plasma.

### Lysosomal enzyme assay

Plasma was separated from 2 ml of peripheral blood from the 16 patients and their parents. 4-Mu-β-D-gluronide (MW:352 g/mol) and 4-Mu-α-D-mannopyronoside (MW:338 g/mol) (Sigma-Aldrich) were used as the fluorogenic substrates to determine lysosomal enzyme activity. The activity was expressed as the amount of substrate (nmol) cleaved per h per ml of protein in the plasma. The normal ranges in the Chinese controls were found to be 10.7–33.7 nmol h-1 per ml protein for β-D-glucuronidase and 13.7–66.7 nmol h-1 per ml protein for α-D-mannosidase.

### Molecular analysis

Genomic DNA was isolated from whole blood obtained from the 16 patients and their parents. DNA isolation was performed using the Qiagen DNA isolation kit (D-5000) (Gentra Systems, Inc., Minneapolis, MN, USA) according to the manufacturer’s protocol.

The full coding exons, their exon-intron boundaries, and the 5’- and 3’-flanking regions of the *GNPTAB* gene were amplified using primers designed according to the published sequence (UCSC NM_024312). Primers and conditions are available upon request. PCR was performed in a 25 μl reaction mixture containing 20 ng of genomic DNA, 2.5 μl of 10× PCR buffer I, 10 mmol dNTPs, 10 pmol each of the forward and reverse primers, and 0.5 U of AmpliTaq (Takara Biotechnology, Dalian, China). Samples were incubated in a thermocycler for 5 minutes at 95°C; followed by 35 cycles of 94°C for 30 seconds, annealing temperature of 58.5°C for 30 seconds and 72°C for 60 seconds; and then a final extension at 72°C for 10 minutes.

The PCR products were sequenced in both directions using an ABI 3730XL sequencer (Biomed Corporation, Beijing, China).

The results were compared with the normal control (UCSC NM_024312). The mutations identified were confirmed by sequencing of the second PCR amplicons. For reference, the A of the ATG translation initiation codon of the coding sequence of *GNPTAB* is referred to as nucleotide +1.

We also analyzed the genomic DNA sequence of *GNPTG* (UCSC NM_032520) in two persons in whom only one mutation in *GNPTAB* was detected. The method used was published in our previous article [12].

## Results

### Clinical phenotype

#### Patients with MLII

The MLII group contains 8 patients (including 5 males and 3 females) ranging in age from 1 to 2.5 years old (Table 1). One patient died when he was 2.5 years old (patient 5). All of the MLII patients had different clinical symptoms and signs since birth, but the time of diagnosis was after 1 year of age. Most patients (2) had been poor feeders since 2 months of age, and 3 patients showed obvious scoliosis after 6 months of age.

**Table 1:** The phenotypes and genotypes in our MLII patients.

Finding/Patients		1	2	3	4	5	6	7	8
General	Gender/Age(years)	M/1.5	M/1	F/2.5	F/2.5	M/2	F/1.5	M/1.5	M/1.5
	Height(cm)	46	73.5	75	75	67	50	50	70
	Weight(kg)	10	7	9	9	8	9	7.5	8
	Age of onset	Birth	2m	5m	5m	6m	7m	6m	6m
	Onset of signs	Feeding difficulties	Feeding difficulties	Feeding difficulties	Feeding difficulties	Kyphosis	Kyphosis	Kyphosis	Finger stiffness
Craniofacial	Coarse face	+	+	+	+	+	+	+	+
	Corneal clouding	+	+	+	+	+	+	+	-
	Gingival hyperplasia	+	+	+	+	+	+	+	+
Skeletal	Claw hand	+	+	+	+	+	+	+	+
	Pectus	+	-	-	-	+	-	+	+
	Joint stiffness	+	+	+	+	+	+	+	+
	Vertebral scoliosis	-	-	-	-	+	+	+	+
	Unaided walking	-	-	-	-	-	-	-	-
Abdomen	Hepatomegaly	+	+	-	-	-	-	-	-
	Splenomegaly	+	+	-	-	-	-	-	-
	Inguinal hernia	+	+	-	-	-	+	+	+
Speech	Speak single words	+	-	+	+	-	-	-	+
Cardiovascular (echocardiacography)	Mitral	N	N	N	N	N	regurgitation	N	N
	Tricuspid	regurgitation	N	N	N	N	N	regurgitation	N
	Aortic	prolapse, regurgitation	N	N	N	N	N	N	N
	Left Atrial	N	N	N	N	N	N	N	N
	Left Ventricular	enlargement	N	N	N	N	N	N	N
Family history	Consanguineous parents	-	-	-	-	-	-	-	-
	Affected sibling	-	-	-	-	-	-	1 brother	-
Activity of GlcNAc-1-phosphotransferase (plasma)	β-D-glucuronidase (10.7–33.7nmol/h/ml)	877.8	694.3	890.7	724.8	816.9	654.2	880.8	818.6
	α-D-mannosidase (13.7–66.7nmol/h/ml)	1006.1	914.5	980.2	956.3	1005.2	857.4	912.3	1008.2
GNPTAB mutation		c.1090C>T (p.R364X)	c.1212C>G (p.Y404X)	c.99delC (p.S33Sfs50X)	c.2455G>T (p.E819X)	c.2213C>A (p.S740X)	c.1071G>A (p.W357X)	c.2821dupA (p.I941Nfs4X)	c.1090C>T (p.R364X)
		c.2550_2554delGAAA (p.K850NfS10X)	c.2715+1G>A	c.2715+1G>A	c.2715+1G>A	c.3613C>T (p.R1205X)	ND	ND	c.1212C>G (p.Y404X)

Prenatal histories documented abnormalities in 6 of 7 pregnancies, including oligohydramnios in 3 cases, intrauterine growth retardation in 2 cases, preterm deliveries in 4 cases, and small for gestational age in 6 cases.

All 8 patients had dysmorphic features, including facial coarseness with depressed nasal bridge, metopic prominence and hypertrophied gums. Corneal clouding appeared as early as 2 months of age.

The patients’ skeletal problems included hand contracture in 8 patients, cubitus valgus in 7 patients, pectus carinatum in 4 patients and vertebral scoliosis in 4 patients. Three probands could sit unsupported, and none could walk unaided at the time of diagnosis. Five patients experienced recurrent respiratory infections, and 3 patients had mitral and tricuspid regurgitation. Hepatosplenomegaly and inguinal hernia were noted in 2 patients.

The MLII patients had delayed neuromotor development. Nearly all of the MLII patients made vocal sounds, but verbal expression remained limited to single words.

#### Patients with MLIII alpha/beta

The MLIII alpha/beta group contains 8 patients (including 3 males and 5 females). The ages of the patients ranged from 4 years to 11 years, and the time period from initial skeletal signs of disease to diagnosis varied from 1 year to 8 years. All patients were admitted to our clinic for progressive joint stiffness for at least one year.

Contractures of the hands were evident between 1 and 6 years of age in all of the patients. Difficulty walking was noticed after the age of 3 in 8 patients. Eight patients complained of shoulder stiffness and loss of flexibility before the age of 6. Decreased range of motion of the wrists was noted by 3 years of age in 4 patients. Severe scoliosis developed in 4 patients (Table 2).

**Table 2:** The phenotypes and genotypes in our MLIII alpha/beta patients.

Finding/Patients		9	10	11	12	13	14	15	16
General	Gender/Age(years)	F/4	M/8	M/11	F/7	F/9	F/7	M/8	F/8
	Height(cm)	90	128.5	139.5	110	128	118	118.5	122.5
	Weight(kg)	14.5	28	27.5	20	21	20	27	23.5
	Age of oneset	1y	4y	3y	3y	3y	2y	1y	6y
	Onset of signs	Pectus	Finger stiffness	Finger and knee stiffness	Finger stiffness	Finger and wrist stiffness	Finger stiffness	Finger stiffness	Finger stiffness
Craniofacial	Coarse face	-	-	-	-	-	-	-	-
	Corneal clouding	-	-	-	-	-	-	-	-
	Gingival hyperplasia	-	-	-	-	-	-	-	-
Skeletal	Claw hand	+	+	+	+	+	+	+	+
	Pectus	+	-	-	-	-	-	-	-
	Joint stiffness	H,S,K	H,E,S	H,W,E,S,K	H,E,S	H,W,E,S	H,S	H,S	H,W,E,S,K
	Vertebral scoliosis	+	-	-	-	-	-	+	+
	Unaided walking	+	+	+	+	+	+	+	+
Abdomen	Hepatomegaly	-	-	-	-	-	-	-	-
	Splenomegaly	-	-	-	-	-	-	-	-
	Inguinal hernia	-	-	-	-	-	-	-	-
Speech	Speak single words	+	+	+	+	+	+	+	+
Cardiovascular (echocardiacography)	Mitral	N	prolapse, regurgitation	thickening	N	prolapse, regurgitation	regurgitation	N	regurgitation
	Tricuspid	N	N	prolapse	N	N	regurgitation	N	N
	Aortic	N	N	thickening	N	N	N	thickening	thickening
	Left Atrial	N	N	N	N	enlargement	enlargement	N	enlargement
	Left Ventricular	N	N	N	N	N	N	N	hypertrophy
Family history	Consanguineous parents	-	-	-	-	-	-	-	-
	Affected sibling	-	-	-	-	-	1 brother	-	1 brother
Activity of GlcNAc-1-phosphotransferase (plasma)	β-D-glucuronidase (10.7–33.7nmol/h/ml)	823.6	684.8	750.4	899.6	714.9	729.4	858.1	726.7
	α-D-mannosidase (13.7–66.7nmol/h/ml)	894.7	816.3	900.8	934.6	894.8	916.2	1143.8	904.7
GNPTAB mutation		c.1090C>T (p.R364X)	c.2715+1G>A	c.1090C>T (p.R364X)	c.525_526del AAinsG (p.K175Kfs12X)	c.118-1G>A	c.2345C>T (p.S782F)	c.2455G>T (p.E819X)	c.2356C>T (p.Q786X)
		c.2345C>T (p.S782F)	c.3136-2A>G	c.2345C>T (p.S782F)	c.2715+1G>A	c.2715+1G>A	c.2715+1G>A	c.2715+1G>A	c.2715+1G>A

Mitral and/or aortic valve regurgitation or thickening was detected in 6 patients. Enlargement of the left atrium was observed in 3 patients. Hypertrophy of the left ventricular wall was observed in 1 patient.

The intelligence levels of the MLIII alpha/beta patients were normal. Two patients received high school diplomas, and 1 patient attended college. All 8 MLIII patients had normal prenatal history, craniofacial features, lung function tests and abdominal ultrasound.

#### Radiographic findings

**Fig 1:**
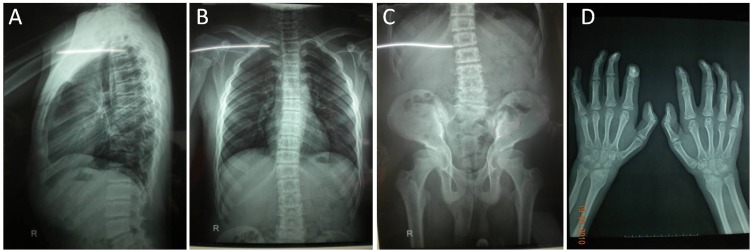
The radiological characteristics of MLIII patients. (A, B) Mild hyperlordosis of the thoracic and lumbar spine and mild irregularities of the upper and lower end plates. Widened ribs, especially in the lateral and frontal (costochondral junction) parts. (C) Joint stiffness of the hip. (D) Diaphyses of the metacarpals and phalanges are nearly normal in length and width.

Radiographic evaluations of all of our patients showed signs of spondyloepiphyseal dysplasia. In MLII patients, the hand tubular bones were significantly shortened, and the metaphyses were extremely widened. The hyperlordosis of the thoracic and lumbar spine and irregularities of the upper and lower end plates were serious. In MLIII alpha/beta patients, the hand tubular bones were of normal or near-normal length. They showed mild generalized platyspondyly, variable vertebral dysplasia and widened ribs (Fig 1).

### Lysosomal enzymes in the plasma

In all affected patients, the plasma activities of two lysosomal hydrolases were significantly increased compared to controls (19-27-fold for β-D-glucuronidase and 12-17-fold for α-D-mannosidase). There was no significant difference in the activity of both lysosomal hydrolases between MLII and MLIII alpha/beta patients.

### Mutations in MLII and MLIII alpha/beta patients

We detected 15 mutations in 30 of 32 alleles in 16 Chinese patients (Tables 1–3). These include 7 nonsense mutations (c.1090C>T, p.R364*; c.1212C>G, p.Y404*; c.2455G>T, p.E819*; c.1071G>A, p.W357*; c.2213C>A, p.S740*; c.2356C>T, p.Q786*; and c.3613C>T, p.R1205*), 1 missense mutation (c.2345C>T, p.S782F), 3 splicing mutations (c.2715+1G>A, c.118-1G>A and c.3136-2A>G) and 4 frameshift mutations (c.99delC, p.S33Sfs50X; c.523_524delAAinsG, p.N175Afs38X; c.2550_2554delGAAA, p.K850Nfs10X; and c.2821dupA, p.I941Nfs4X). Ten of these mutations (67%) (Table 2) had not been previously reported. The most frequent mutations were c.2715+1G>A, c.1090C>T and c.2345C>T, accounting for 28% (9/32), 13% (4/32) and 9% (3/32), respectively, of all of the mutations observed in our patients. The majority of the mutations are located in exon 13 or in its exon-intron junctions where 57% (17/30) of the mutant alleles were identified. No mutation was found in *GNPTG* in 2 patients in whom only one *GNPTAB* mutation was detected (Table 3).

**Table 3:** Pathogenic *GNPTAB* gene mutation summary in our 16 patients.

	Alteration	Location	Effect	Alleles	Frequency	Phenotype
1	c.2715+1G>A	I13	Splicing	9	28.13%	MLII,MLIII
2	c.1090C>T	E9	p.R364*	4	12.50%	MLII,MLIII
3	**c.2345C>T**	E13	p.S782F	3	9.38%	MLIII
4	**c.1212C>G**	E10	p.Y404*	2	6.25%	MLII,MLIII
5	**c.2455G>T**	E13	p.E819*	2	6.25%	MLII,MLIII
6	**c.99delC**	E1	p.S33Sfs50X	1	3.13%	MLII
7	**c.118-1G>A**	I1	Splicing	1	3.13%	MLIII
8	**c.523_524delAAinsG**	E5	p.N175Afs38X	1	3.13%	MLIII
9	c.1071G>A	E9	p.W357*	1	3.13%	MLII
10	**c.2213C>A**	E13	p.S740*	1	3.13%	MLII
11	**c.2356C>T**	E13	p.Q786*	1	3.13%	MLIII
12	c.2550_2554delGAAA	E13	p.K850Nfs10X	1	3.13%	MLII
13	**c.2821dupA**	E14	p.I941Nfs4X	1	3.13%	MLII
14	**c.3136-2A>G**	I15	Splicing	1	3.13%	MLIII
15	c.3613C>T	E20	p.R1205*	1	3.13%	MLII

Bold letters indicate new mutations in this study

## Discussion

Prior to December, 2015, a total of 145 mutations in the *GNPTAB* gene had been reported, including 60 (41%) missense and nonsense mutations, 36 (25%) small deletion mutations, 28 (19%) small insertion mutations and 15 (10%) splicing mutations. The most frequent mutation is the deletion c.3503_3504delTC (p.L1168QfsX5) that was detected in all 27 MLII obligatory carriers in French Canada [13], in 51% of 38 Italian MLII and MLIII alpha/beta patients [14], and in 22% of 61 MLII and MLIII alpha/beta patients in the United States [8]. In addition, this mutation was found worldwide and resulted most probably from an unique founder molecular lesion [15].

In Asia, the nonsense mutation c.3565C>T (p.R1189*) was found to be the most frequent mutation in 40 (allele frequency 41%) Japanese patients [16]. The mutations c.3565C>T (p.R1189*) and c.2715+1G>A were detected in 5 (allele frequency 50%) unrelated Korean patients [17].

Since 2010, 4 ML II Chinese families, including 1 Taiwanese family have been analyzed[18–20]. Five mutations were reported in these families, including c.2715+1G>A; c.1090C>T, p.R364*; c.1071G>A, p.W357*; c.2422delC, p.L808fs19X; and c.3565C>T, p.R1189*. Of the 15 mutations detected in our patients, the mutations c.2715+1G>A; c.1090C>T, p.R364* and c.1071G>A, p.W357* were previously reported in Asian patients, but 10 mutations were novel. The majority of the mutant alleles (53%) were found in exon 13 or in its intronic flanking regions. In opposition to what has been described for the majority of the populations already studied, in which the c.3503_3504delTC is the most frequent mutation, in Chinese patients the most common mutation is the splicing mutation c.2715+1G>A (allele frequency 28%). This could be related to the existence of a mutational hot spot in that area of the gene, or, most likely, the high frequency of this splicing mutation was due to a founder effect. The other 5 mutations found in the same gene region (exon 13 and surrounding introns) were rare, and a plausible explanation for their high number may simply be the length of exon 13 (1103 bp), which is the longest exon of the gene (3771 bp).

We detected 3 different splicing mutations. The mutation c.2715+1G>A had also been reported in the Japanese and Korean populations and was associated with reduced GlcNAc-1-phosphotransferase activity and an attenuated ML phenotype [16,21]. In 2005, Paik et al. performed RT-PCR analysis, which indicated the skipping of exon 13 in the presence of this mutation [21].Both novel mutations, c.118-1G>A in intron 1 and c.3136-2A>G in intron 15 occur at splicing acceptor sites. Because of the lack of access to fresh peripheral blood of patients to extract mRNA, we did not perform an RT-PCR analysis. However, we can speculate that these splicing mutations may originate transcripts that either contain a non-spliced intron or a skipped exon, as a result of aberrant splicing. To assess its potential effect on mRNA splicing, the relative strengths of the 5’ and 3’ splice-site signals were evaluated using the Neural Network (NN) splice prediction program (available at www.fruitfly.org/seq_tools/splice.html). The score value predicted by the software for both wild type splice sites is 0.99 (normal range from 0 to 1). The presence of both mutations reduces the score values to less than 0.

According to the previous literature, the nonsense and frameshift mutations resulted in truncated phosphotransferases that are retained in the ER and cannot be activated by the site-1 protease in the Golgi apparatus [22–24]. Consequently, all of these mutations lead to MLII (at least in homozygosity). We speculate that the pathogenic effects of our 7 nonsense and 4 frameshift mutations were the same.

We found one missense mutation, c.2345C>T (p.S782F), in 3 MLIII alpha/beta patients. To determine whether the new mutation was a polymorphism, we analyzed exon 13 of the *GNPTAB* gene in 50 healthy Chinese individuals, and we did not find the same nucleotide change at this site. We also analysed the mutation through the basic prediction software programs Polyphen (http://genetics.bwh.harvard.edu/pph2/index.shtml) and SIFT (http://blocks.fhcrc.org/sift/SIFT.html). The c.2345C>T alteration was classified as likely to be damaging and not tolerated, which was consistent with a high likelihood of pathogenicity. The S782F mutation is located in the DMAP domain of the α subunit, which was shown to be responsible for the recognition of lysosomal enzymes [25].

In previous studies, the MLII phenotype was considered to be directly correlated with the *GNPTAB* genotypes. Homozygotes or compound heterozygotes were expected to produce no or nearly no gene product, and ‘null’ or “amorph” alleles were associated with nonsense and frameshift mutations, irrespective of the mutational location. The presence of at least one hypomorph, missense or splicing allele in the *GNPTAB* genotype resulted in the clinically milder MLIII [8, 22 26,27]. However, De pace et al. recently found that both missense (p.Trp81Leu) and frameshift mutations that were associated with a severe clinical phenotype caused retention of the encoded protein in the endoplasmic reticulum and failure to cleave the α/β subunit precursor protein in ML patients [23]. Based on the mutations detected in our 8 MLII patients, including nonsense, frameshift and splicing mutations, we speculate these are associated with a severe clinical phenotype.

In 2009, Takanobu Otomo et al. concluded that the nonsense mutations c.310C>T (p.Q104*), c.2681G>A (p.W894*) and c.3565C>T (p.R1189*) were associated with clinically severe phenotypes in 40 Japanese MLII and III patients [16]. However, in our patients, we did not detect those three nonsense mutations. In our study, 6 of 8 MLII patients had nonsense mutations. Four of seven nonsense mutations identified in our patients were found only in the MLII patients, and 2 nonsense mutations were found in MLII and MLIII alpha/beta patients. The patients #5 and #8, who were carrying 2 nonsense mutations both exhibited severe phenotypes, supporting the previous results that the severe phenotypes are associated with the presence of nonsense mutations.

The mutations c.1071G>A(p.W357*), c.1090C>T(p.R364*), c.3613C>T(p.R1205*), and c.2550_2554delGAAA had all previously been found in the homozygous state in MLII patients[8,14,22]. Therefore, by principle, these mutations were expected to be responsible for more severe forms of MLII. Our patients #1, #5, and #6 were compound heterozygotes for those three mutations in combination with either nonsense or frameshift mutations and all had MLII. However, patients #9 and #11 with the same genotype of c.1090C>T in combination with the missense mutation c.2345C>T had attenuate MLIII. The c.2345C>T mutation is located in the DMAP domain, and Qian et al. found that two other missense mutations in the DMAP domain (K732N and L785W) exhibited reduced GlcNAc-1-phosphotransferase activity of 20%[28].

In 2009, S S Cathey et al. analyzed 61 ML patients and detected 7 splicing mutations, which were all found in MLIII patients [23]. Takanobu Otomo et al. studied 40 ML patients and found only 1 splicing mutation (c.2715+1G>A) in MLIII patients [16]. In this study, 3 splicing mutations were detected. Two novel mutations, c.118-1G>A and c.3136-2A>G, were found in 2 separate MLIII alpha/beta patients. These two mutations may be associated with a mild clinical phenotype. The most frequent mutation among Chinese patients, the splicing mutation c.2715+1G>A, which was expected to determine an attenuate MLIII phenotype (Paik et al. 2005), was found in both severe MLII (3 patients) and attenuate MLIII (6 patients) forms in combination with other mutations. Interestingly, the same genotype p.E819*; c.2715+1G>A was observed in patients #4 (MLII, disease onset at 5 months) and #15 (MLIII, disease onset at 1 year). Our data first reported the association between the c. 2715+1G>A mutation and MLII. We speculated that it could be due to the alternative splicing effect caused by this mutation in different patients, and it could also be due to the existence of other regulatory genes, potentially reversing the “beneficial” effect of c.2715+1G>A. In the future, more samples will be required to investigate the relationship between the genotype and phenotype of the ML patients with splicing mutations.

The clinical diagnoses of MLII and MLIII are based on the clinical manifestations, skeletal abnormalities and activity analyses of several lysosomal enzymes in the plasma. The majority of MLII patients are diagnosed based on typical facial features or family history. Fatal outcomes often occur before 10 years of age, and cardiorespiratory failure that is refractory to treatment is the cause of death in most affected children [29]. MLIII is a slowly progressive inborn error of metabolism that mainly affects the skeletal, joint and connective tissues, and the patients have the ability to survive to adulthood. Although the clinical severity among patients with MLII is more extensive than MLIII, there was no significant difference in enzyme activity between them. The genetic bases of MLII and MLIII alpha/beta should depend on *GNPTAB* gene mutation screening.

This is the most comprehensive molecular analysis of Chinese ML patients. We reported 15 mutations, including 10 novel mutations, in 16 patients. We found that c.2715+1G>A is the most common mutation in our patients. In addition, the majority of the mutations reported in the Chinese patients were located on exon 13 or in its intronic flanking regions. Our data revealed genotype-phenotype correlations in Chinese MLII and MLIII alpha/beta patients.

## Supporting Information

S1 File Mutations of our ML patients. (PDF)

## References

[1] Kornfeld S, Sly W (2001) I-cell disease and pseudo-Hurler polydystrophy: disorders of lysosomal enzyme phosphorylation and localization The metabolic and molecular bases of inherited disease, 8th edn McGraw-Hill, New York: 3469–3482.

[2] Cathey S. S., Kudo M., Tiede S., Raas-Rothschild A., Braulke T., et al (2008). Molecular order in mucolipidosis II and III nomenclature. American journal of medical genetics. Part A, 146(4), 512. 10.1002/ajmg.a.32193 18203164

[3] Bao M., Booth J. L., Elmendorf B. J., Canfield W. M. (1996). Bovine UDP-N-acetylglucosamine: Lysosomal-enzyme N-Acetylglucosamine-1-phosphotransferase I PURIFICATION AND SUBUNIT STRUCTURE. Journal of Biological Chemistry, 271(49), 31437–31445. 8940155 10.1074/jbc.271.49.31437

[4] Kudo M, Bao M, D'Souza A, Ying F, Pan H, et al (2005) The alpha- and beta-subunits of the human UDP-N-acetylglucosamine:lysosomal enzyme N-acetylglucosamine-1-phosphotransferase [corrected] are encoded by a single cDNA. J Biol Chem 280: 36141–36149. 16120602 10.1074/jbc.M509008200

[5] Tiede S., Storch S., Lübke T., Henrissat B., Bargal R., Raas-Rothschild A, et al (2005). Mucolipidosis II is caused by mutations in GNPTA encoding the α/β GlcNAc-1-phosphotransferase. Nature medicine, 11(10), 1109–1112. 16200072 10.1038/nm1305

[6] Raas-Rothschild A, Cormier-Daire V, Bao M, Genin E, Salomon R, Brewer K., et al (2000) Molecular basis of variant pseudo-hurler polydystrophy (mucolipidosis IIIC). J Clin Invest 105: 673–681. 10712439 10.1172/JCI5826 PMC289169

[7] Marschner K., Kollmann K., Schweizer M., Braulke T., Pohl S. (2011). A key enzyme in the biogenesis of lysosomes is a protease that regulates cholesterol metabolism. Science, 333(6038), 87–90. 10.1126/science.1205677 21719679

[8] Cathey SS, Leroy JG, Wood T, Eaves K, Simensen RJ, Kudo M., et al (2010) Phenotype and genotype in mucolipidoses II and III alpha/beta: a study of 61 probands. J Med Genet 47: 38–48. 10.1136/jmg.2009.067736 19617216 PMC3712854

[9] Umehara F., Matsumoto W., Kuriyama M., Sukegawa K., Gasa S., Osame M, et al (1997). Mucolipidosis III (pseudo-Hurler polydystrophy); clinical studies in aged patients in one family. Journal of the neurological sciences, 146(2), 167–172. 9077513 10.1016/s0022-510x(96)00301-2

[10] Pohl S, Tiede S, Castrichini M, Cantz M, Gieselmann V, Braulke T, et al (2009) Compensatory expression of human N-acetylglucosaminyl-1-phosphotransferase subunits in mucolipidosis type III gamma. Biochim Biophys Acta 1792: 221–225. 10.1016/j.bbadis.2009.01.009 19708128

[11] Zarghooni M, Dittakavi SS (2009) Molecular analysis of cell lines from patients with mucolipidosis II and mucolipidosis III. Am J Med Genet A 149A: 2753–2761. 10.1002/ajmg.a.33134 19938078

[12] Liu S, Zhang W, Shi H, Meng Y, Qiu Z (2014) Three novel homozygous mutations in the GNPTG gene that cause mucolipidosis type III gamma. Gene 535: 294–298. 10.1016/j.gene.2013.11.010 24316125

[13] Plante M, Claveau S, Lepage P, Lavoie EM, Brunet S, Roquis D., et al (2008) Mucolipidosis II: a single causal mutation in the N-acetylglucosamine-1-phosphotransferase gene (GNPTAB) in a French Canadian founder population. Clin Genet 73: 236–244. 10.1111/j.1399-0004.2007.00954.x 18190596

[14] Tappino B, Chuzhanova NA, Regis S, Dardis A, Corsolini F, Stroppiano M., et al (2009) Molecular characterization of 22 novel UDP-N-acetylglucosamine-1-phosphate transferase alpha- and beta-subunit (GNPTAB) gene mutations causing mucolipidosis types IIalpha/beta and IIIalpha/beta in 46 patients. Hum Mutat 30: E956–973. 10.1002/humu.21099 19634183

[15] Coutinho M. F., Encarnação M., Gomes R., da Silva Santos L., Martins, Sirois‐Gagnon D., et al (2011). Origin and spread of a common deletion causing mucolipidosis type II: insights from patterns of haplotypic diversity. Clinical genetics, 80(3), 273–280. 10.1111/j.1399-0004.2010.01539.x 20880125

[16] Otomo T, Muramatsu T, Yorifuji T, Okuyama T, Nakabayashi H, Fukao T., et al (2009) Mucolipidosis II and III alpha/beta: mutation analysis of 40 Japanese patients showed genotype-phenotype correlation. J Hum Genet 54: 145–151. 10.1038/jhg.2009.3 19197337

[17] Paik KH, Song SM, Ki CS, Yu HW, Kim JS, Min K. H., et al (2005) Identification of mutations in the GNPTA (MGC4170) gene coding for GlcNAc-phosphotransferase alpha/beta subunits in Korean patients with mucolipidosis type II or type IIIA. Hum Mutat 26: 308–314. 16116615 10.1002/humu.20205

[18] Yang Y, Wu J, Liu H, Chen XH, Wang Y, Zhao MC et al (2013). Two homozygous nonsense mutations of GNPTAB gene in two Chinese families with mucolipidosis II alpha/beta using targeted next-generation sequencing. Genomics, 102(3): 169–173. 10.1016/j.ygeno.2013.06.001 23773965

[19] Ma G C, Ke Y Y, Chang S P, Lee D J, Chen M. (2011). A compound heterozygous GNPTAB mutation causes mucolipidosis II with marked hair color change in a Han Chinese baby. American Journal of Medical Genetics Part A, 155(4): 931–934. 10.1002/ajmg.a.33834 21416587

[20] Zhan T, Cui X, Xing X, Ren A, Gan G, Liu Y, et al (2011). Mucolipidosis in a Chinese family with compound heterozygous mutations at the GNPTAB gene. Clinica Chimica Acta, 412(15): 1469–1471. 10.1016/j.cca.2011.04.025 21549105

[21] Heo JS, Choi KY, Sohn SH, Kim C, Kim YJ, Shin S. H, et al (2012) A case of mucolipidosis II presenting with prenatal skeletal dysplasia and severe secondary hyperparathyroidism at birth. Korean J Pediatr 55: 438–444. 10.3345/kjp.2012.55.11.438 23227064 PMC3510274

[22] Tiede S, Storch S, Lubke T, Henrissat B, Bargal R, Raas-Rothschild A, et al (2005) Mucolipidosis II is caused by mutations in GNPTA encoding the alpha/beta GlcNAc-1-phosphotransferase. Nat Med 11: 1109–1112. 16200072 10.1038/nm1305

[23] Pace R., Coutinho M. F., Koch‐Nolte F., Haag F., Prata M. J., Alves S., et al (2014). Mucolipidosis II‐related mutations inhibit the exit from the endoplasmic Reticulum and proteolytic cleavage of GlcNAc‐1‐phosphotransferase precursor protein (GNPTAB). Human mutation, 35(3), 368–376. 10.1002/humu.22502 24375680

[24] Velho R. V., De Pace R., Klünder S., Sperb-Ludwig F., Lourenço C. M., Schwartz I. V. et al (2015). Analyses of disease-related GNPTAB mutations define a novel GlcNAc-1-phosphotransferase interaction domain and an alternative site-1 protease cleavage site. Human molecular genetics, 24(12), 3497–3505. 10.1093/hmg/ddv100 25788519 PMC4498157

[25] Qian Y., Flanagan-Steet H., van Meel E., Steet R., Kornfeld S. A. (2013). The DMAP interaction domain of UDP-GlcNAc: lysosomal enzyme N-acetylglucosamine-1-phosphotransferase is a substrate recognition module. Proceedings of the National Academy of Sciences, 110(25), 10246–10251. 10.1073/pnas.1308453110 PMC3690890 23733939

[26] Kudo M, Brem MS, Canfield WM (2006) Mucolipidosis II (I-cell disease) and mucolipidosis IIIA (classical pseudo-hurler polydystrophy) are caused by mutations in the GlcNAc-phosphotransferase alpha / beta -subunits precursor gene. Am J Hum Genet 78: 451–463. 16465621 10.1086/500849 PMC1380288

[27] Bargal R, Zeigler M, Abu-Libdeh B, Zuri V, Mandel H, Neriah Z. B., et al (2006) When Mucolipidosis III meets Mucolipidosis II: GNPTA gene mutations in 24 patients. Molecular Genetics And Metabolism 88: 359–363. 16630736 10.1016/j.ymgme.2006.03.003

[28] Qian Y., van Meel E., Flanagan-Steet H., Yox A., Steet R., Kornfeld S. (2015). Analysis of mucolipidosis II/III GNPTAB missense mutations identifies domains of UDP-GlcNAc: lysosomal enzyme GlcNAc-1-phosphotransferase involved in catalytic function and lysosomal enzyme recognition. Journal of Biological Chemistry, 290(5), 3045–3056. 10.1074/jbc.M114.612507 25505245 PMC4317033

[29] Pagon, R. A., Adam, M. P., Ardinger, H. H., Bird, T. D., Dolan, C. R., Bean, L. J. H., et al. (2012). Mucolipidosis II. GeneReviews® [Internet]. Available from: http://ezproxy.kmu.edu.tw:2060/books/NBK1828/

