# Supplementary material for: Mutation Analysis of 16 Mucolipidosis II and III Alpha/Beta Chinese Children Revealed Genotype-Phenotype Correlations
Source: PLoS One. 2016 Sep 23;11(9):e0163204. doi: 10.1371/journal.pone.0163204 (PMC5035076; doi:10.1371/journal.pone.0163204)

The mutations of ML patients

Total number:15

c.1090C>T

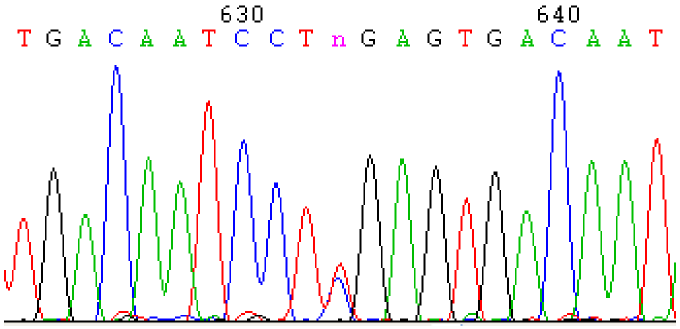

c.2541-2545delAAAAG

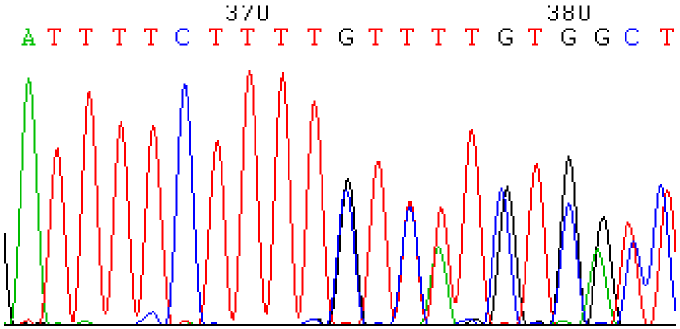

c.1212C>G

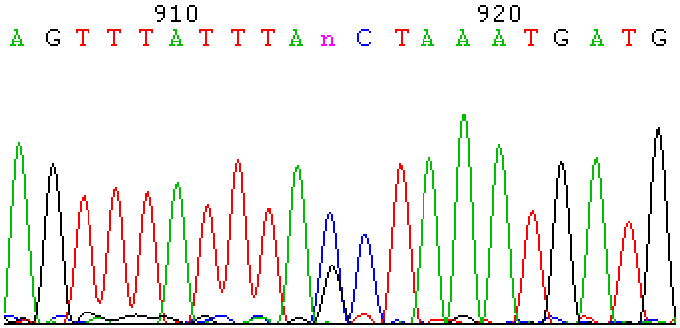

c.2715+1G>A

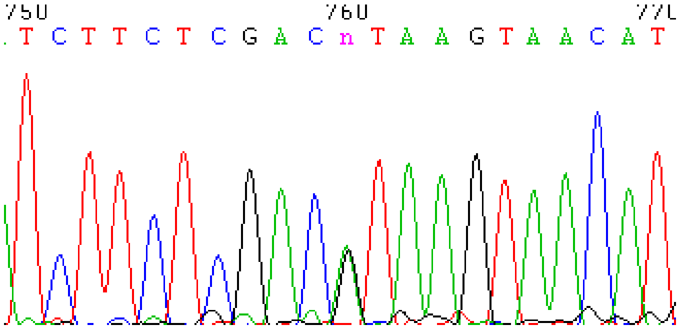

c.98delC

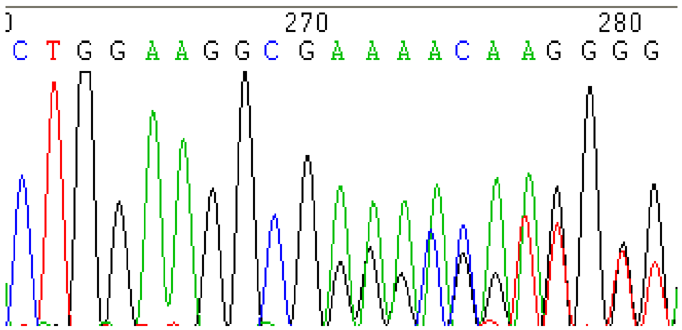

c.2455G>T

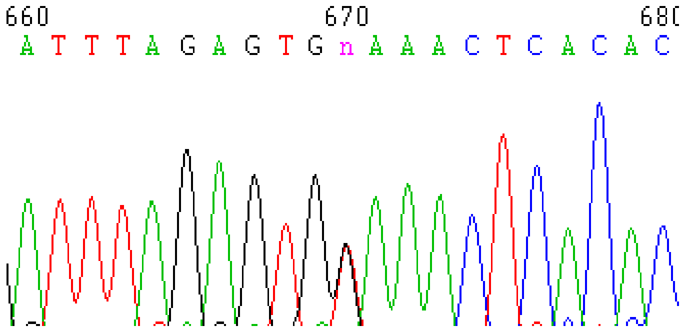

c.2219C>A

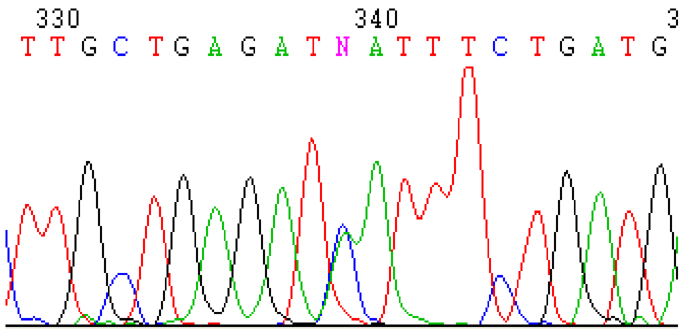

c.3613C>T

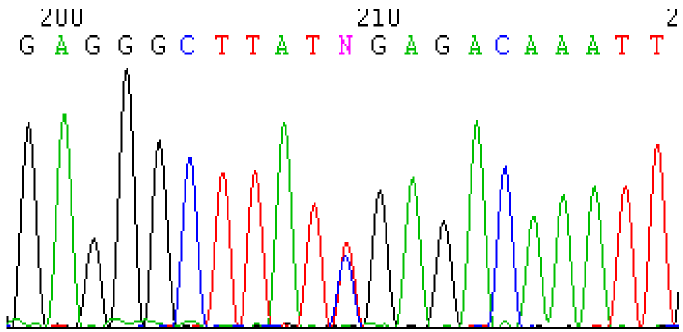

c.1071G>A

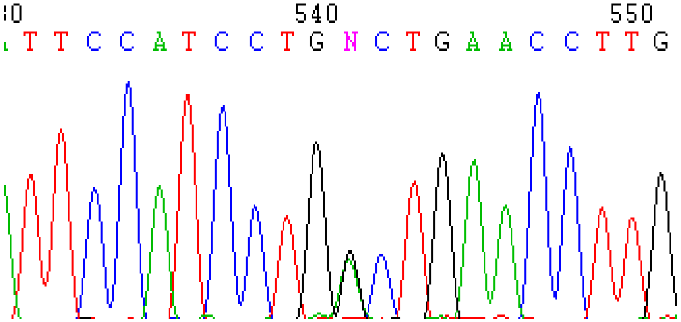

c.2822dupA

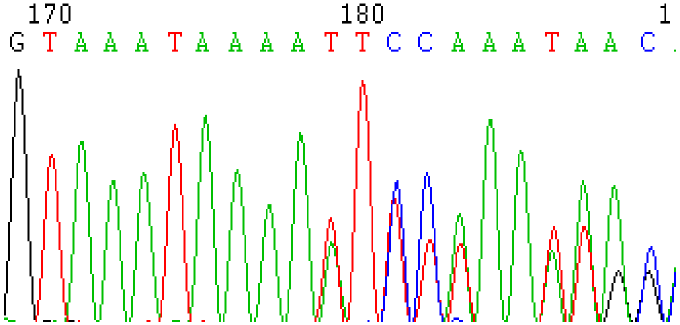

c.3136-2A>G

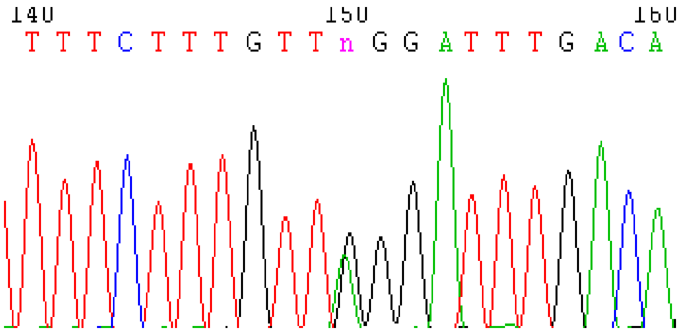

c.523_525delAAinsG

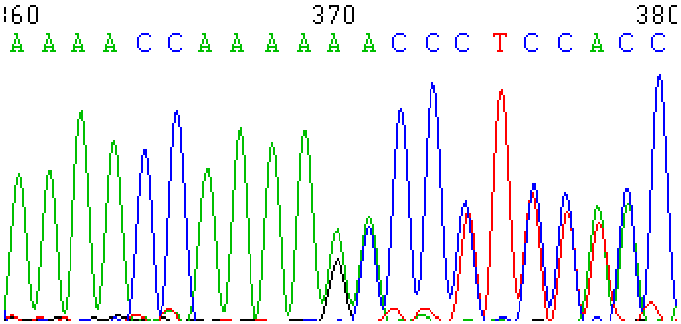

c.118-1G>A

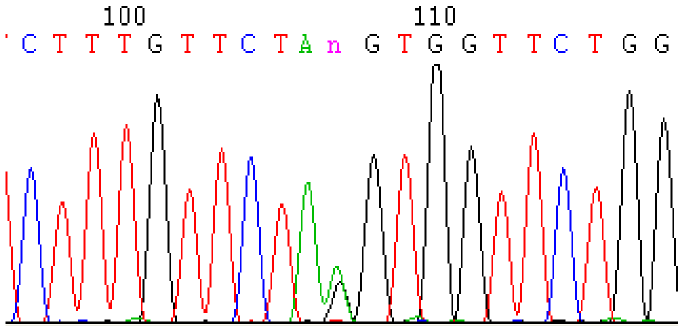

c.2345C>T

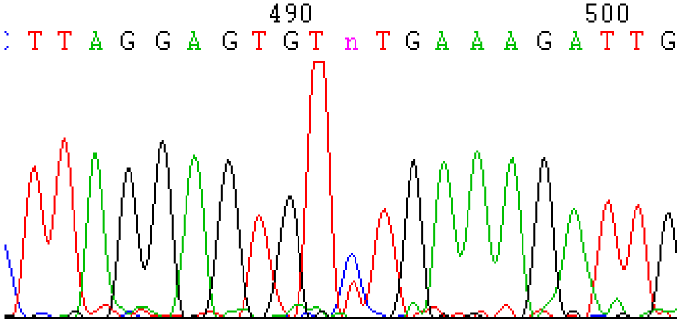

c.2356C>T

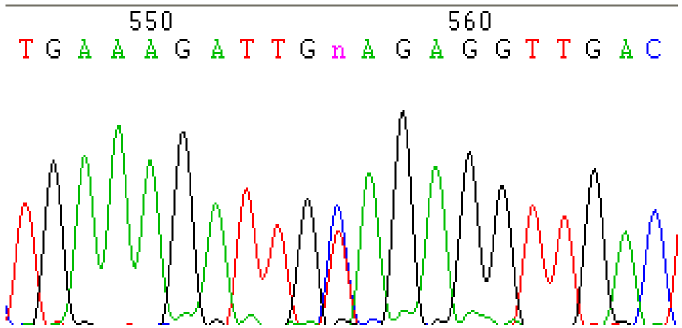

Supplement: S1 File — (PDF) [file pone.0163204.s001.pdf]
